# GBDT-Based Fall Detection with Comprehensive Data from Posture Sensor and Human Skeleton Extraction

**DOI:** 10.1155/2020/8887340

**Published:** 2020-06-25

**Authors:** Wen-Yu Cai, Jia-Hao Guo, Mei-Yan Zhang, Zhi-Xiang Ruan, Xue-Chen Zheng, Shuai-Shuai Lv

**Affiliations:** ^1^College of Electronics and Information, Hangzhou Dianzi University, Hangzhou 310018, China; ^2^Zhejiang Provincial Key Lab of Equipment Electronics, Hangzhou Dianzi University, Hangzhou 310018, China; ^3^School of Electrical Engineering, Zhejiang University of Water Resources and Electric Power, Hangzhou 310018, China

## Abstract

Since fall is happening with increasing frequency, it has been a major public health problem in an aging society. There are considerable demands to distinguish fall down events of seniors with the characteristics of accurate detection and real-time alarm. However, some daily activities are erroneously signaled as falls and there are too many false alarms in actual application. In order to resolve this problem, this paper designs and implements a comprehensive fall detection framework on the basis of inertial posture sensors and surveillance cameras. In the proposed system framework, data sources representing behavior characteristics to indicate potential fall are derived from wearable triaxial accelerometers and monitoring videos of surveillance cameras. Moreover, the NB-IoT based communication mode is adopted to transmit wearable sensory data to the Internet for subsequent analysis. Furthermore, a Gradient Boosting Decision Tree (GBDT) classifier-based fall detection algorithm (GBDT-FD in short) with comprehensive data fusion of posture sensor and human video skeleton is proposed to improve detection accuracy. Experimental results verify the good performance of the proposed GBDT-FD algorithm compared to six kinds of existing fall detection algorithms, including SVM-based fall detection, NN-based fall detection, etc. Finally, we implement the proposed integrated systems including wearable posture sensors and monitoring software on the Cloud Server.

## 1. Introduction

An increased aging population in the world is forcing rapid rises in healthcare requirements [1]. Everyone knows that older people have poor balance ability and slow response ability. Falls are a major cause of injury for the elderly and a huge obstacle in the independent living of the seniors. Once the elderly falls down alone at home without help, the injured elderly may be lying on the ground for several hours or even days. More seriously, it is very likely to extended injury and be life-threatening if he did not get treatment timely. Therefore, timely fall incident detection and medical assistance for the elderly are intuitively important. However, due to different application scenarios and various body activities, satisfactory and reliable fall detection results are too hard to guarantee [2].

Some related fall detection algorithms have been proposed in the literature. Broadley et al. [3] review the latest reported systems on activity monitoring of humans based on wearable sensors and issues to be addressed to tackle the challenges. As far as we know, there are three main categories of fall detection technologies: fall detection using wearable sensors [4, 5], fall detection using environmental sensors [6, 7], and video-based fall detection [8, 9]. Although there are some other methods such as radar-based fall detection [10], they are more complicated compared to the above three methods.

Wearable sensor based fall detection methods mainly depend on sensory data gathered from wearable accelerometer and gyroscope. It is generally agreed that the use of wearable sensors has played a quite important role in monitoring the physiological parameters of a person to minimize any malfunctioning happening in the body. In recent years, the advancement of sensing technologies, embedded systems, wireless communication technologies, nanotechnologies, and miniaturization makes it possible to develop smart wearable sensors to monitor activities of human beings continuously. Nag et al. [11] provide a review on some of the significant research work done on wearable flexible sensors. Chen et al. [12] propose a novel intelligent fall detection method, named as ESAEs-OCCCH, which uses acceleration data from a wrist-worn smart watch. ESAEs-OCCCH is first adopted for unsupervised feature extraction to overcome the disadvantages of artificial feature extraction. Yacchirema et al. [13] propose an innovative IoT (Internet of Thing) based online system for detecting falls of the aged. Sensory readings are processed and analyzed using a decision tree based Big Data model running on a Smart IoT Gateway [14]. Although these wearable sensors have high sensitivity and good real-time characteristics, higher detection accuracy cannot typically be achieved due to the interference from diverse activities of hand or wrist. Hence, it is easy to cause misjudgment and missed detection of fall actions relying only on wearable sensory data.

Secondly, a few scholars apply environmental sensors to detect falls. Li et al. [15] propose a phase transform (SRP-PHAT) method which can locate the original source of a certain voice. In terms of sound classification phase, they apply the Mel-Frequency Cepstral Coefficients (MFCC) features with a Nearest Neighbor (NN) approach to improve fall detection performance. However, expensive acoustic devices have high requirements on the environment, and it is not feasible to promote accurate detection with certain ambient noise. Adnan et al. [16] adopt acoustic Local Ternary Patterns (acoustic-LTPs) to detect fall events by analyzing environmental noise. Acoustic features are extracted from the separated source components using the proposed acoustic-LTPs scheme. Subsequently, fall events would be identified with SVM based classifier. However, it will cause noise during the audio signal acquisition, which could lead to low accuracy and frequent false alarms.

Thirdly, vision-based fall detection typically uses image processing techniques to construct a human body model to detect fall. In general, video-based fall detection systems have shown some potential and reliability in detecting falls in public places. Due to the popularity of video surveillance, vision-based fall detection methods have already become one research hotspot. The boundary extraction method is used to obtain the aspect ratio of the human body and then to judge falls. Sase and Bhandari [17] used contour-based template matching to distinguish human and nonhuman and then judged human fall according to the distance between the top and center of the external rectangle of the human body. Shen et al. [18] propose a fall detection method using the Deeper Cut model to exact human key points, and it is implemented using Raspberry Pi platform. Vision-based fall detection can use relatively cheap cameras to quantify and judge various activities; nevertheless, it requires complex handling methods to construct a human body model and it is unsuitable for real-time detection mode.

In addition, some results have suggested that a single detection model from individual DataSet could easily lead to false detection. Recently, there are already a few methods on the basis of wearable sensors and surveillance cameras to classify body activities. Kepski and Kwolek [19] apply a Kinect camera and a device consisting of an accelerometer and a gyroscope, and then a fuzzy inference system is used to separate fall from daily activities. Hondori et al. [20] present a detection system that helps monitor various dining activities of poststroke patients using a Kinect camera and an accelerometer. Nizam et al. [21] propose a novel approach that uses a depth sensor and employs a unique procedure that identifies the fall risk levels to adapt the algorithm for different people with their physical strength to withstand falls. Bogdan et al. [22] present a low-cost system for reliable fall detection with a very low false alarm ratio on the basis of accelerometric data and depth maps. The single drawback of the above methods is that Kinect camera is not cheap since a considerable computational power is needed to execute image processing algorithms. He et al. [23] propose a method to integrate the information of video images, sound, infrared, pulse, and other information into the elderly care system. However, it is not very realistic to detect fall accidents with so many sensors.

To overcome these shortcomings, we use an ordinary camera and accelerometer as a data source in this paper so as to improve the practicability of the detection system. Furthermore, a novel data fusion based fall detection online system and one GBDT based detection algorithm is provided in detail. Data fusion of human activity features obtained by posture sensor and surveillance cameras plays a significant role in the recognition of abnormal activities. In addition, the proposed platform uses NB-IoT communication and Ethernet to transmit manifold data to the Cloud Server for further analysis. The platform is able to effectively monitor the daily life of the elderly. When an unexpected fall incident occurs, the proposed system will send an alarm signal to inform the family relatives or other related guardians. In conclusion, the proposed system would meet the requirements of high sensitivity and precision. As a result, necessary assistance could be provided in times with high coverage communication technology, so it is suitable for application in the elderly care system.

The rest of the paper is organized as follows.  Section 2 describes the whole online fall detection platform framework and comprehensive data source. In Section 3, GBDT based fall detection algorithm using comprehensive data from an accelerometer based posture sensor and human skeleton extraction is presented in detail. Comparable experimental results and actual operating interface are described in Section 4. Finally, a conclusion is drawn in Section 5.

## 2. System Framework and Comprehensive Data Source

**Figure 1:**
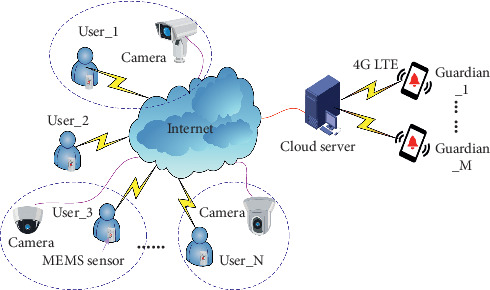
Framework of the proposed platform.

**Figure 2:**
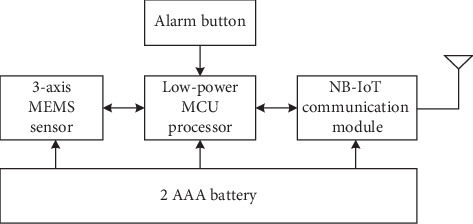
The block diagram of self-made posture sensor.

Our complete framework of the fall detection system for seniors is illustrated in Figure 1. Each user is equipped with a kind of self-made MEMS (Micro Electro Mechanical Systems) based wearable sensor with hardware block diagram in Figure 2, which uses triaxial acceleration and angular velocity sensor to capture the body posture. Besides, NB-IoT (Narrow Band Internet of Things) [24] communication mode is used to transmit sensory data to the Cloud Server. With the development of Internet of Things technology, the health care field is also affected deeply. As we know, NB-IoT is an emerging technology with many good features such as wide coverage, multiple connections, low speed, low power consumption, etc. In our IoT based health monitoring system, various detection devices are connected together for data exchange, so as to deliver warnings to medical staff or guardians in time when the elderly fall. Moreover, it is also supposed that each user is covered by at least one surveillance camera and so we can monitor and record each user's activity. Today, almost all surveillance cameras have the ability to transmit video sequences to the Internet through Ethernet or wireless networks. Therefore, the Cloud Server could obtain both attitude data and video data and store it at the local database for further analysis. The fall detection algorithm is running on the Cloud Server with high performance. Once fall events are detected, the Cloud Server will send an alarm signal to the specific guardian through 4G LTE (Long Term Evolution) communication technology. As a result, each user can get instant help and timely treatment in case of any abnormality with our proposed framework.

**Figure 3:**
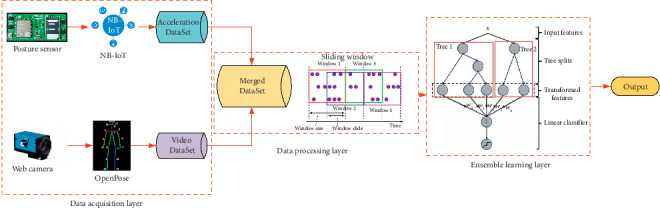
The principle of GBDT-FD algorithm.

The principle of the fall detection process is demonstrated in Figure 3. In this system framework, real-time acceleration data from posture sensors are transmitted by NB-IoT communication mode to the Cloud Server, and then form a data collection named as Acceleration DataSet (ADS). Surveillance cameras are used to collect human activities' video, and key point coordinates of human body are obtained through OpenPose software [25] processing to form Video DataSet (VDS). Video DataSet and Acceleration DataSet together make up the so-called Merged DataSet (MDS). After processing by sliding window strategy [26], one ensemble learning method named Gradient Boost Decision Tree (GBDT) [27] is applied for self-learning with MDS, so as to classify fall and other normal activities in a robust way.

Since human activity frequency generally does not exceed 20 Hz [28], the acceleration acquisition frequency is set to 30 Hz so as to process data more accurately, and video acquisition frequency is adjusted to 30 fps (frames per second) after software processing. The entire self-made DataSet comprises six kinds of human activities involving fall, walk, sit, squat, lie down, and jump.

Due to the diversity and complexity of fall accidents, it is hard to identify the way and direction of fall events. To overcome this shortcoming, we use the sum vector of triaxial acceleration value to measure the human activity. Let *a*_*x*_, *a*_*y*_, *a*_*z*_ denote acceleration value in three dimensions, respectively; *A*_3-axis_ represent the actual value of triaxial acceleration, which can be calculated with the following equation:

(1) $$\begin{array}{c} A_{3-\text{axis}}=\sqrt{a_{x}^{2}+a_{y}^{2}+a_{z}^{2}}. \end{array}$$

**Figure 4:**
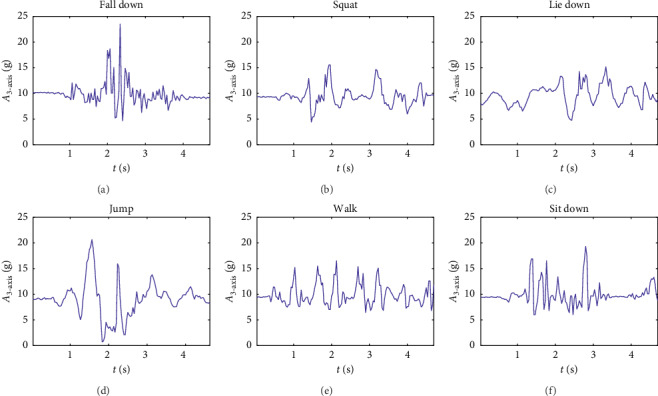
*A*_3*-*axis_ variation with different human activities.

The measured sensory data are illustrated in Figure 4 to compare the acceleration variation curve of falls and that of other normal activities, including squat, lie down, jump, walk, and sit down.

**Figure 5:**
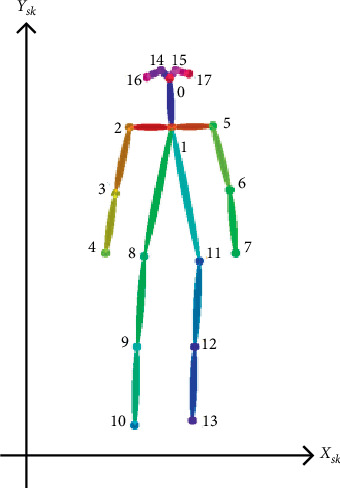
Skeleton scattergram of human body.

The other fall characteristics are key skeleton coordinates of the human body as shown in Figure 5. The rectangular coordinate system is established in Figure 5, and the horizontal and vertical coordinates of *N* = 18 key points would be obtained, respectively. (*X*_*sk*−*i*_, *Y*_*sk*−*i*_) (*i* = 1, 2,…, *N*) denotes the respective coordinates of each key point. Hence, the collection of key skeleton coordinates is as follows:

(2) $$\begin{array}{c} \left(X_{sk},Y_{sk}\right)=\left\{\left(X_{sk-1},Y_{sk-1}\right),\left(X_{sk-2},Y_{sk-2}\right),\ldots ,\left(X_{sk-18},Y_{sk-18}\right)\right\}. \end{array}$$

However, the total 18 key points extracted using OpenPose software are not all effective and necessary for identifying fall events. Redundant data will only increase computation complexity and even introduce unnecessary noises. Therefore, the maximum and minimum *x*-coordinate and *y*-coordinate of each key skeleton point *X*_*sk*−max_, *X*_*sk*−min_, *Y*_*sk*−max_ and *Y*_*sk*−min_ are picked out, respectively, and then a body circumscribed rectangle is introduced to represent the contour of the human body. The behavior change of the human body can be identified only by paying attention to the changes of length and width of body rectangle, so as to reduce calculation complexity significantly compared to that using all the key skeleton points.

**Figure 6:**
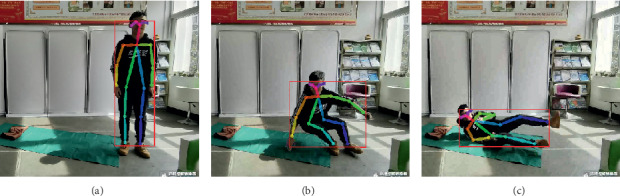
Three different scenarios during a fall process. (a) Standing. (b) Losing balance. (c) Falling down.

Figure 6 illustrates three different scenarios of standing, losing balance, and falling down completely. The colored rectangle denotes the outline of the human body, and it is very clear that the length and width of the body rectangle have changed a lot. We regard the aspect ratio *R* between body width and length as a feature from Video DataSet, which is calculated as follows:

(3) $$\begin{array}{c} R=\frac{X_{sk-\max}-X_{sk-\min}}{Y_{sk-\max}-Y_{sk-\min}}. \end{array}$$

**Figure 7:**
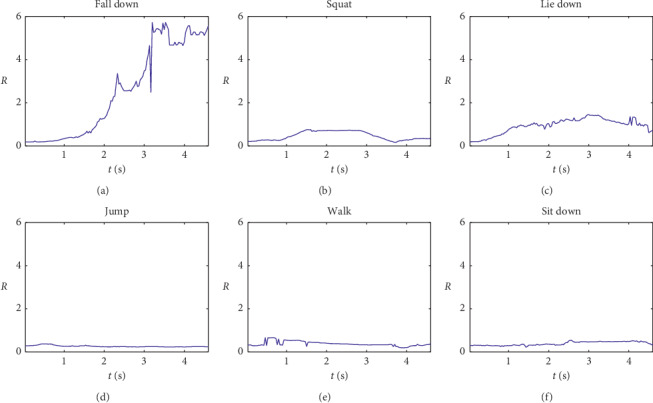
Aspect ratio *R* variation with different human activities.

Due to complex surroundings in different monitoring scenarios, sometimes there is no guarantee that OpenPose software can obtain 18 key points completely. In this case, the median of the previous occurrence of this point and the next occurrence of this point will be used instead.  Figure 7 compares the aspect ratio *R* between falls and other normal activities.

**Figure 8:**
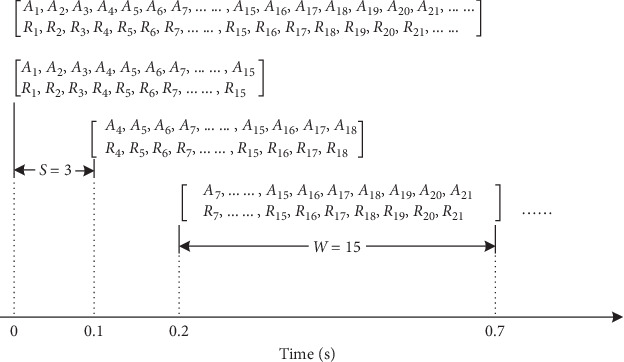
Sliding window.

Since the fall process is a continuous dynamic event in the time dimension, it could not be identified by acceleration data or video data in a moment. In order to search and detect the complete process of the falling event, we use a sliding window method to intercept the derived data flow as Figure 8, where *A* and *R* denote the sum vector of triaxial acceleration value and the aspect ratio of the human body, respectively. Following the study in Ref. [29], the complete fall process ranges from 0.3 s to 0.4 s, so we set width value and step value to 0.5 s and 0.1 s, respectively. In our GBDT-FD algorithm, the data acquisition frequency is 30 Hz, so there are *W* = 15 pieces of data in a 0.5 s sliding window, and the step value between each two data sequences is *S* = 3 pieces of data.

After obtaining *A*_3-axis_ and *R*, it is necessary to extract the statistical characteristic so as to carry out the classification process. When fall incident occurs, the acceleration and posture of the human body will exceed normal range and change rapidly. In conclusion, we should only pay attention to the overall size and change rate of *A*_3*-*axis_ and *R* within each sliding window. For both Acceleration DataSet and Video DataSet, 7 characteristic values including mean, standard deviation (std), maximum value (max), minimum value (min), average change rate (*d*), number of mean crossings (MCV), and root mean square (RMS) of each sliding window are calculated, respectively. MCV means the number of samples above the mean value in a set of data, so too large or too small MCV means this dataset change too dramatically. As a result, the above 14-dimensional statistical characteristics are listed in Table 1.

We extracted 14-dimensional statistical characteristics from each sliding window to constitute the Merged DataSet, which is stored as in the following matrix:

(4) $$\begin{array}{c} \left[\begin{array}{c} A_{\text{mean}1},A_{\text{std}1},A_{\max1},A_{\min1},A_{d1},A_{\text{MCV}1},A_{\text{RMS}1},R_{\text{mean}1},R_{\text{std}1},R_{\max1},R_{\min1},R_{d1},R_{\text{MCV}1},R_{\text{RMS}1}\\ A_{\text{mean}2},A_{\text{std}2},A_{\max2},A_{\min2},A_{d2},A_{\text{MCV}2},A_{\text{RMS}2},R_{\text{mean}2},R_{\text{std}2},R_{\max2},R_{\min2},R_{d2},R_{\text{MCV}2},R_{\text{RMS}2}\\ A_{\text{mean}3},A_{\text{std}3},A_{\max3},A_{\min3},A_{d3},A_{\text{MCV}3},A_{\text{RMS}3},R_{\text{mean}3},R_{\text{std}3},R_{\max3},R_{\min3},R_{d3},R_{\text{MCV}3},R_{\text{RMS}3}\\ \ldots \\ A_{\text{mean}i},A_{\text{std}i},A_{\max i},A_{\min i},A_{di},A_{\text{MCV}i},A_{\text{RMS}i},R_{\text{mean}i},R_{\text{std}i},R_{\max i},R_{\min i},R_{di},R_{\text{MCV}i},R_{\text{RMS}i} \end{array}\right], \end{array}$$

where subscript *i* denotes that this characteristic sequence is extracted from the *i*-th sliding window.

## 3. GBDT-Based Fall Detection Algorithm

### 3.1. Ensemble Learning-Based Fall Detection

**Figure 9:**
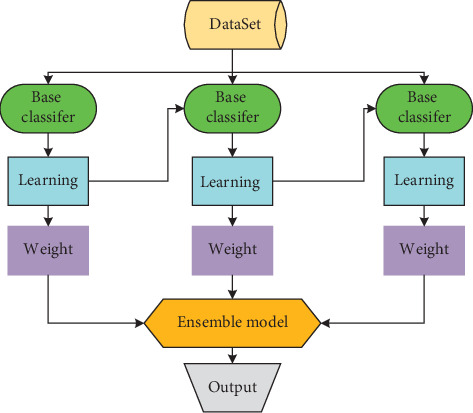
Ensemble learning.

Ensemble learning [30] is a machine learning method that combines multiple weak learners into a strong one. Several individual learners with complementary results are trained concurrently, and the results from each individual learner are merged into the final prediction result by a certain combination strategy, so as to achieve the effect of extensive learning and effective classification. If each individual learner is the same type in ensemble learning, these individual learners are called base learners. The advantage of ensemble learning lies in its strong robustness, which enables it to reduce the interference of noisy data effectively. Moreover, normalization is not required in the data preprocessing stage. Gradient boosting is a machine learning technique for regression problems, which produces a prediction model in the form of an ensemble of weak prediction models. GBDT (Gradient Boosting Decision Tree) is a boosting method using Classification and Regression Tree (CART) [31] as its base learner which is illustrated in Figure 9. GBDT uses decision tree as the weak prediction model in gradient boosting so it has high accuracy.

The described residual is the difference between the correct result and the actual result. In GBDT-FD algorithm, we use the logarithmic loss function to define residuals as follows:

(5) $$\begin{array}{c} L\left(y,f\left(X\right)\right)=\log\left(1+\exp\left(-yf\left(X\right)\right)\right). \end{array}$$

The 14-dimensional Merged DataSet MDS defined in Section 2 is as follows:

(6) $$\begin{array}{c} \text{MDS}=\left\{\left(X_{1},y_{1}\right),\left(X_{2},y_{2}\right),\ldots ,\left(X_{m},y_{m}\right)\right\}, \end{array}$$

where *X* denotes 14-dimensional data, which is shown as follows: *y* denotes labels, *y*=1 means fall, and *y*=−1 means normal activity:

(7) $$\begin{array}{c} X=\left(A_{\text{mean}},A_{\text{std}},A_{\max},A_{\min},A_{d},A_{N},A_{\text{RMS}},R_{\text{mean}},R_{\text{std}},R_{\max},R_{\min},R_{d},R_{N},R_{\text{RMS}}\right). \end{array}$$

The optimal parameters of the GBDT model-based fall detection algorithm are determined by the evaluation method with *k*-fold cross-validation, which means that the whole DataSet is divided into *k* mutually exclusive subsets with approximately equal size. One subset is taken as the test set and the rest as the training set while training GBDT model. In this way, the model can be trained and tested for *k* times to evaluate the performance of the model more objectively.

GBDT classifier is regarded as an additional model composed of CART [31] illustrated in equation (8). Classification is realized by continuously decreasing the residuals generated in the training process as follows:

(8) $$\begin{array}{c} f\left(X\right)=\sum_{n=1}^{N}h\left(X;\theta_{n}\right), \end{array}$$

where *f*(*X*) denotes the overall model, *h*(*X*; *θ*_*n*_) denotes the *n*-th decision tree, *θ*_*n*_ denote parameters of the *n*-th decision tree, *N* denotes the number of decision trees.

The purpose of GBDT is to make loss function reduce as fast as possible and preferably fall along its gradient direction. At each round, the negative gradient of the log-likelihood loss function is used to fit the new CART, which could accelerate the reduction and convergence of loss function as soon as possible and finally speed up the training process. GBDT-FD will train CART for fall detection through several iterations.

The following describes the main steps of the GBDT-FD algorithm.

#### 3.1.1. Initialization

Firstly, GBDT-FD algorithm chooses one feature from *X* as the CART node and then selects an appropriate eigenvalue as the segmentation point. For example, *A*_mean_ is taken as the root node. In general, *A*_mean_ value of a fall is usually much larger than that of normal activity. Finally, the Merged DataSet is divided into two categories with the eigenvalue boundary of preset segmentation. It can be roughly considered that these two categories represent falls and normal activities, respectively, but the loss function will be very large. GBDT-FD will iterate through all combinations of features and segmentation points to minimize the loss function of CART. Therefore, initial CART has only one root node as in the following equation and *M* denotes the number of samples:

(9) $$\begin{array}{c} f_{0}\left(X\right)=\arg\min\sum_{m=1}^{M}L\left(y_{m},c\right). \end{array}$$

#### 3.1.2. Iterations

Suppose the number of iterations is *T*, and *e*_*tm*_ denotes the negative gradient error of *m*-th sample in the *t*-round iteration; hence,

(10) $$\begin{array}{c} e_{tm}=-{\left[\frac{\partial L\left(y_{m},f\left(X_{m}\right)\right)}{\partial f\left(x_{m}\right)}\right]}_{f\left(X\right)=f_{t-1}\left(X\right)}=\frac{y_{m}}{1+\exp\left(y_{m}f\left(x_{m}\right)\right)}. \end{array}$$

GBDT-FD will train the CART of this iteration according to (*X*_*m*_, *e*_*tm*_)(*i*=1,2,…, *M*). Leaf node region *Re*_*tj*_(*j*=1,2,…, *J*) will be obtained by *J* denoting the number of regression leaf nodes. Using linear search, estimate the value of the leaf node region and minimize the loss function:

(11) $$\begin{array}{c} c_{tj}=\arg\min\sum_{X_{m}\in Re_{tj}}\log\left(1+\exp\left(-y_{m}\left(f_{t-1}\left(X_{m}\right)+c\right)\right)\right). \end{array}$$

Since equation (11) is difficult to optimize, we use approximation as follows:

(12) $$\begin{array}{c} c_{tj}=\frac{\sum_{X_{m}\in Re_{tj}}e_{tm}}{\sum_{X_{m}\in Re_{tj}}\left|e_{tm}\right|\left(1-\left|e_{tm}\right|\right)}. \end{array}$$

Then a new learner generated by this iteration process can be expressed as follows:

(13) $$\begin{array}{c} f_{t}\left(X\right)=f_{t-1}\left(X\right)+\sum_{j=1}^{J}c_{tj}I,\;X_{m}\in Re_{tj}. \end{array}$$

However, in order to avoid overfitting, learning rate *v* ranging in (0,1] should be considered when updating learners, so the evolution rule of a new learner is as follows:

(14) $$\begin{array}{c} f_{t}\left(X\right)=f_{t-1}\left(X\right)+v\cdot \sum_{j=1}^{J}c_{tj}I,\;X_{m}\in Re_{tj}. \end{array}$$

In general, much more iterations mean smaller loss function and better performance. However, the loss function will decrease less obviously as the number of iterations increases. In order to save training time and computational resources, GBDT-FD sets training termination conditions:

The training process will stop if,

(15) $$\begin{array}{c} \Delta L\left(y,f\left(X\right)\right)<\varepsilon , \end{array}$$

where Δ*L*(*y*, *f*(*X*)) denotes the decrease of the loss function, and *ε* is the training termination threshold set by the GBDT-FD algorithm.

#### 3.1.3. Classification

GBDT-FD classification process is as follows:

1. Input unclassified test dataset
2. The trained CART will start from the root node and compare with the input data based on features and segmentation points
3. After the comparison process is completed, CART will output the category of each input data
4. Test datasets are classified into two categories: fall and nonfall

### 3.2. Performance Metrics

As we know, for the dichotomous problem, the final classifying results can be divided into four categories: True Positive samples (TP), False Negative samples (FN), False Positive samples (FP), and True Negative samples (TN) on the basis of different combinations of real category and predicted category, which are summarized in Table 2.

Since fall detection belongs to a typical dichotomous problem (fall and nonfall), five integrated metrics are used to verify performance usually [2].

1. Precision: the ratio between real falling samples and predicted falling samples:

(16) $$\begin{array}{c} I_{\text{pre}}=\frac{\text{TP}}{\text{TP}+\text{FP}}. \end{array}$$

(2) Sensitivity: the ratio between samples correctly identified as falls and real falling samples:

(17) $$\begin{array}{c} I_{\text{sens}}=\frac{\text{TP}}{\text{TP}+\text{FN}}. \end{array}$$

(3) Specificity: the ratio between samples correctly identified as nonfalls and real nonfalling samples:

(18) $$\begin{array}{c} I_{\text{spec}}=\frac{\text{TN}}{\text{TN}+\text{FP}}. \end{array}$$

(4) Accuracy: the ratio between samples correctly identified and the total sample set:

(19) $$\begin{array}{c} I_{\text{acc}}=\frac{\text{TP}+\text{TN}}{\text{TP}+\text{FN}+\text{TN}+\text{FP}}. \end{array}$$

(5) *F*-score: weighted harmonic mean of precision and sensitivity:

(20) $$\begin{array}{c} I_{\beta -\text{score}}=\frac{\left(\beta^{2}+1\right)\cdot I_{\text{pre}}\cdot I_{\text{sens}}}{\beta^{2}\cdot I_{\text{pre}}+I_{\text{sens}}}, \end{array}$$

where *β* is a nonnegative parameter, and different *β* indicates different weights between precision and sensitivity. Apparently, there are three main cases:

1. *β*=1, denoting the weight of *I*_pre_ and *I*_sens_ is equal
2. *β* > 1, denoting the weight of *I*_sens_ is greater than that of *I*_pre_
3. *β* < 1, denoting the weight of *I*_pre_ is greater than that of *I*_sens_

According to the difference of *β*, *I*_1_-score, *I*_0.5_-score, and *I*_2_-score are commonly used in statistical occasions. In terms of fall detection, we should pay attention to the sensitivity of the detection model so as to reduce health hazards effectively, and moreover, ensure the wounded could get immediate medical assistance when fall occurs. Meanwhile, wrong fall alarm may lead to a waste of communication resource, so it is necessary to pay attention to the precision of the model, but it is not as important as the sensitivity metric.

## 4. Experimental Results

### 4.1. Experimental Setup

**Figure 10:**
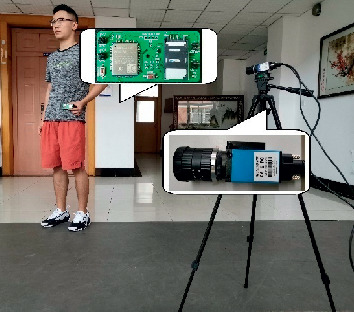
Experimental scene.

The experimental scene at our school is illustrated in Figure 10. Ten graduate students (6 male and 4 female) with average age 26 are volunteered for this research testing. Each user's real-time acceleration data during daily activities is gathered by our wearable posture sensor, and monitoring video is recorded using a surveillance camera. In order to imitate the real behavior of the aged, we also tie sandbags on our feet, so there is amount of falling down slowly situations in our training data set. With this scenario, we got more than 150 videos with each lasting for 1–1.5 minutes. Finally, more than 6000 activities records are used to verify our GBDT based algorithm, 20% of them are used for training, and the rest 80% is used for testing. For safety's sake, the experimental environment is padded completely and tight. The acceleration data and video data are combined to form the merged data to store in Cloud Server and finally input to GBDT based fall down classifier.

Firstly, how to determine optimal parameters for GBDT based fall detection algorithm is discussed. We treat *I*_2_-score and accuracy as main evaluation criteria and *I*_1_-score and *I*_0.5_-score as the reference when training GBDT based detection dichotomy model.

**Figure 11:**
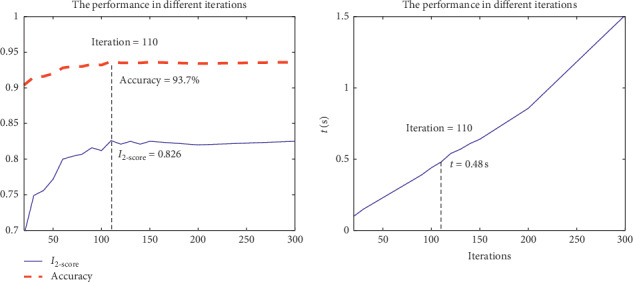
The influence of iteration times of GBDT-FD.

In the whole GBDT model, the iteration times have an important influence on the quality of the model. When the iteration times increase from 20 to 300, the *I*_2_-score and accuracy value of the GBDT model will increase. However, it is clear from Figure 11 when the iteration times exceed 110, *I*_2_-score and accuracy of GDBT model no longer increase significantly. But we can find from Figure 11 that the time required for the training model is still approximately linear, so the optimal value of iteration times is set to 110 in this paper.

Some main parameters of GBDT-FD are shown in Table 3.

### 4.2. Experimental Results

**Figure 12:**
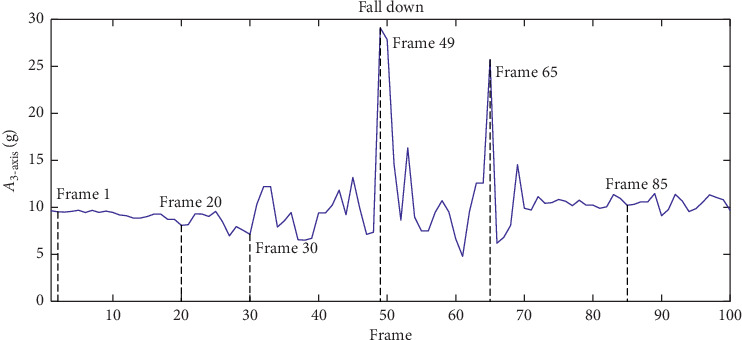
*A*_3−axis_ variation when falling down.

The original sensory data of body activities in a typical fall case are illustrated in Table 4 and Figure 12. Table 4 displays a complete fall process with 6 key frames. Figure 12 demonstrates the detailed change law of the *A*_3-axis_ during this process. It is clear that both types of acceleration sensory data and human posture perception data can reflect the fall process on many occasions.

**Figure 13:**
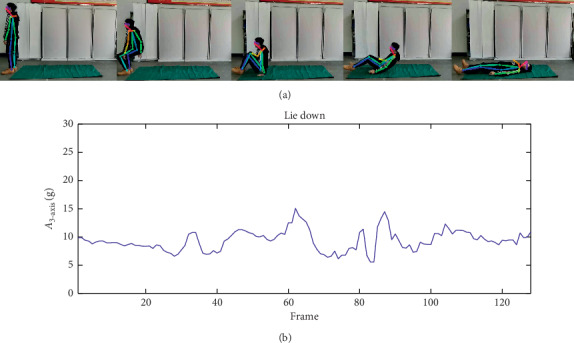
Sensory data when lying down. (a) Video skeleton data when lying down. (b) A3-axis variation when lying down.

Unfortunately, the previous situation is not always guaranteed. In some cases, only a single type of sensory data is difficult to classify falls and normal behaviors accurately. When a user lies down, the aspect ratio *R* will change a lot as given in Figure 13(a), so it will be recognized as fall activity using separated video data. But it is a misjudgment clearly and we can verify this from *A*_3−axis_ variation curve. Nevertheless, the fall classification result of this case should be right using the accelerometric data, which is displayed in Figure 13(b).

**Figure 14:**
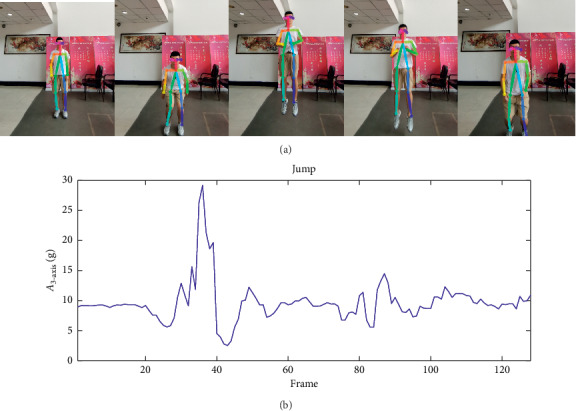
Sensory data when jumping up. (a) Video skeleton data when jumping up. (b) A3-axis variation when jumping up.

In either case, when a user jumps up, it is possible to identify this kind of activity as fall because the acceleration value changes extremely violently. But it is not a fall through video data from Figure 14 because there is no great change of aspect ratio *R*. To summarize, these two special examples verify that more accurate fall detection results could be achieved with data fusion using the Merged DataSet, and not from a single dataset. Hence, this discovery is also the main purpose and significance of this article.

**Figure 15:**
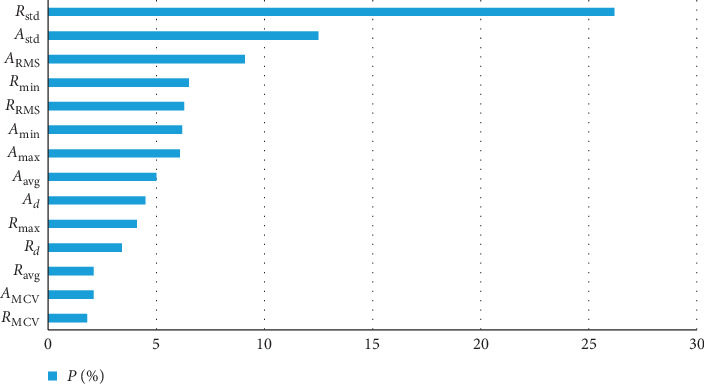
Feature importance in GBDT-FD algorithm.

Secondly, we compare the detection accuracy influence of each feature. GBDT can output the relative importance of each feature to model training so as to help understand the influence of each feature on fall down detection. As shown in Figure 15, the sum of importance ratio of features in Video DataSet (VDS) is 50.4%, and that in Acceleration DataSet (ADS) is 49.6%. This indicates that both datasets play an important role in classification, so we choose all these characteristics as our source data in GBDT-FD.

Furthermore, we compare the fall judgment results of support vector machine (SVM) based Fall Detection (SVM-FD) [32], Naive Bayes (NB) based Fall Detection (NB-FD) [33], Decision Tree (DT) based Fall Detection (DT-FD) [34], *K*-Nearest Neighbor (KNN) based Fall Detection (KNN-FD) [35], Neural Network (NN) based Fall Detection (NN-FD) [36], Random Forest (RF) based Fall Detection (RF-FD) [37], and GBDT based Fall Detection (GDBT-FD) with the Merged DataSet, acceleration DataSet, and skeleton DataSet, respectively. The results in Tables 5–7 explain the comparable detection results of these algorithms with three kinds of DataSet, respectively.

It is clear from Table 5 that *I*_2_-score and accuracy of GBDT-FD are, respectively, 0.878 and 95%. The results of GBDT based fall detection algorithm outperform that of SVM-FD, NB-FD, DT-FD, KNN-FD, NN-FD, and RF-FD, which indicates that GBDT-FD can identify fall events accurately. NN-FD and RF-FD are slightly worse than GBDT-FD. Due to the simplicity of the Naive Bayes model, the performance of the NB-FD algorithm is not ideal too. The Accuracy of SVM-FD and KNN-FD is not much worse than that of GBDT-FD, but there is a big gap between them in *I*_2_-score.

From detection results in Tables 6 and 7 with independent DataSet, it is clear that GBDT-FD has good generalization ability and can handle various types of DataSet. Moreover, the recognition results with Video DataSet are slightly better than that of Acceleration DataSet. This is because the characteristics of video skeleton data are more directly perceived than that of acceleration. However, the performance is still not good as that with Merged DataSet since the Merged DataSet expands the data dimensions, and more features are used to make the model much easier to train. The results verify that using a posture sensor and video skeleton fusion will be more accurate than the traditional individual detection method. However, the other methods have poor generalization ability due to the defects of algorithms. Therefore, the detection results are not satisfactory.

**Figure 16:**
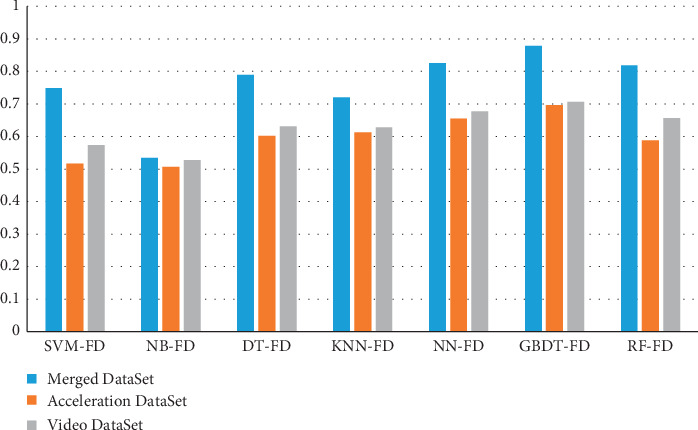
*I*_2_-score comparison of each algorithm with different DataSets.

**Figure 17:**
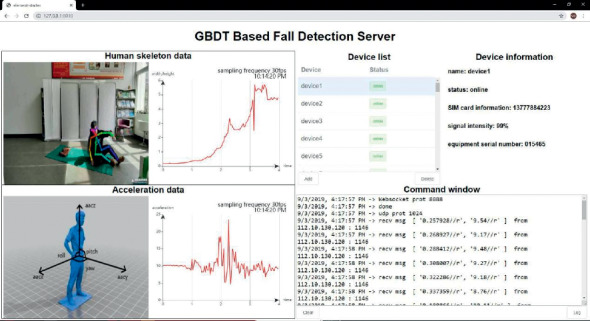
Web interface on the cloud server.

Finally, the most important index *I*_2_-score of each algorithm using three different DataSet is compared in Figure 16. The performance advantages of the proposed GBDT-FD algorithm are obvious. From the aspect of system implementation, the Web interface is shown in Figure 17. There are a few users who have already connected and transmitted sensory data to the Cloud Server. GDBT-FD algorithm is executed on the Server. At present, the well-designed fall detection online platform is already in trial operation in nursing homes, and so it is with a good result in application and worth popularization.

## 5. Conclusions

In this paper, we propose one kind of comprehensive framework of the fall detection system using inertial triaxial acceleration sensors and monitoring cameras to detect fall accidents. The wearable triaxial accelerometer is used to detect the body's posture, and a monitoring camera is used to extract key points of human skeletons information. The fall detection is operated on the basis of fusion based data including accelerometric data and human skeleton key points. In order to reduce false positives of falling incidents, GBDT classifier based fall detection algorithm is investigated in depth. The good performance of the proposed GBDT-FD algorithm is compared with SVM-FD, NB-FD, DT-FD, KNN-FD, NN-FD, and RF-FD in terms of *I*_0.5-score,_*I*_1-score,_*I*_2-score,_ and accuracy, so as to verify the performance improvements of GBDT-FD. In our future work, multiview human skeleton extraction will be adopted, and detection speed will be improved so as to enhance the real-time performance of our system.

## Figures and Tables

**Table 1:** Statistical characteristics.

Acceleration data		Skeleton data	
Features	Symbol	Features	Symbol
Mean value	*A*_mean_	Mean value	*R*_mean_
Standard deviation value	*A*_std_	Standard deviation value	*R*_std_
Maximum value	*A*_max_	Maximum value	*R*_max_
Minimum value	*A*_min_	Minimum value	*R*_min_
Average change value	*A*_*d*_	Average change value	*R*_*d*_
Mean crossings value	*A*_MCV_	Mean crossings value	*R*_MCV_
Root mean square value	*A*_RMS_	Root mean square value	*R*_RMS_

**Table 2:** Different combinations of real category and predicted category.

Real category	Predicted category	
	Fall	Non-fall
Fall	TP	FN
Non-fall	FP	TN

**Table 3:** GBDT-FD parameters.

Parameter	Symbol	Optimal value
Learning rate	*v*	0.35
Iteration rounds	*T*	110
Cross validation	*k*	5
Total sample number	*M*	5160
Terminal condition	*ɛ*	10^−4^

**Table 4:** Fall process.

Frame no.	Scene
1	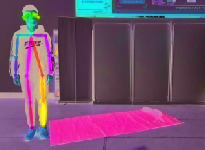
20	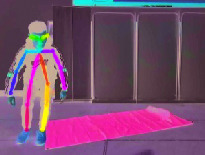
30	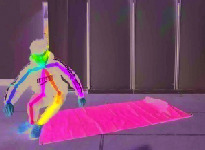
49	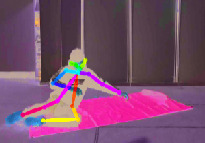
65	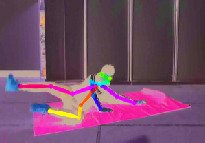
85	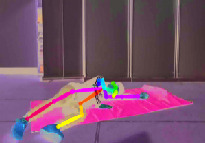

**Table 5:** Detection results of each algorithm with merged DataSet.

Merged DataSet				
Model	*I*_0.5_-score	*I*_1_-score	*I*_2_-score	Accuracy (%)
SVM-FD	0.842	0.793	0.749	91.6
NB-FD	0.630	0.579	0.535	83.5
DT-FD	0.806	0.805	0.789	90.9
KNN-FD	0.785	0.751	0.72	89.7
NN-FD	0.846	0.835	0.825	92.8
RF-FD	0.846	0.831	0.818	92.7
**GBDT-FD**	**0.878**	**0.886**	**0.878**	**95**

**Table 6:** Detection results of each algorithm with Acceleration DataSet.

Acceleration DataSet				
Model	*I*_0.5_-score	*I*_1_-score	*I*_2_-score	Accuracy (%)
SVM-FD	0.707	0.600	0.516	85.8
NB-FD	0.619	0.557	0.506	83.1
DT-FD	0.659	0.613	0.602	83.8
KNN-FD	0.707	0.656	0.613	86.3
NN-FD	0.685	0.670	0.655	85.8
RF-FD	0.741	0.656	0.588	87.1
**GBDT-FD**	**0.777**	**0.737**	**0.696**	**89.2**

**Table 7:** Detection results of each algorithm with Video DataSet.

Video DataSet				
Model	*I*_0.5_-score	*I*_1_-score	*I*_2_-score	Accuracy (%)
SVM-FD	0.728	0.639	0.574	86.6
NB-FD	0.608	0.587	0.567	82.6
DT-FD	0.678	0.677	0.631	84.6
KNN-FD	0.692	0.658	0.628	85.9
NN-FD	0.707	0.692	0.677	86.7
RF-FD	0.739	0.695	0.656	87.6
**GBDT-FD**	**0.754**	**0.728**	**0.706**	**88.6**

## Data Availability

The data supporting the results of this study are available from the corresponding author upon request.
